# Timed relay contact closure controlled system for parallel second dimensions in multi-dimensional liquid chromatography

**DOI:** 10.1186/s13104-019-4506-7

**Published:** 2019-08-01

**Authors:** William Craig Byrdwell

**Affiliations:** Food Composition and Methods Development Lab, Beltsville Human Nutrition Research Center, USDA, ARS, 10300 Baltimore Ave., Building 161, Beltsville, MD 20705 USA

**Keywords:** 2D-LC–MS, APCI-MS, ESI-MS, APPI-MS, Contact closure

## Abstract

**Objective:**

Short-chain triacylglycerols (TAGs) in lipid extracts of biological samples are not sufficiently resolved using conventional reversed-phase separation on two C18 columns in series, or using a two-dimensional chromatographic separation with a silver ion column as the second dimension (^2^D). An additional dimension of separation was required.

**Results:**

The hardware and software components to allow a second second-dimension (^2^D) separation and three total separation dimensions were developed. Two contact closure (CC) activated 4-port, 2-position valves (4P2PVs) for ultra-high performance liquid chromatography (UHPLC) were joined together and used for one of two second dimensions in comprehensive two-dimensional liquid chromatography (2D-LC) coupled to four mass spectrometers simultaneously in parallel in an LC1MS2 × (LC1MS1 + LC1MS1) = LC3MS4 configuration. A timed contact closure circuit (TCCC) controlled the two UHPLC valves, operated by repetitive CCs for the 4P2PVs. The TCCC-controlled 4P2PVs were used to direct a portion of the ^1^D eluent to one of the two ^2^D’s for separation by a quaternary UHPLC system that was not allowed by the commercial 2D-LC system. The ^1^D separation was a non-aqueous reversed-phase HPLC instrument used for separation of TAGs; the commercial 2D-LC ^2^D binary UHPLC was used for silver-ion chromatography of unsaturated TAGs; and the CC-controlled second ^2^D was used for separation of short-chain (SC) saturated TAGs.

## Introduction

Instruments for comprehensive two-dimensional liquid chromatography (2D-LC) are now routinely available. *Comprehensive* 2D-LC produces a separation on a first-dimension (^1^D) column and all or a portion of the effluent is directed to a second-dimension (^2^D) column, with every peak in the ^1^D transferred to the ^2^D column, in contrast to heart-cutting and other 2D-LC approaches. However, there are limitations inherent in commercially available 2D-LC instruments. For instance, the 2D-LC system was designed for the use of one ^2^D binary pump ultra high performance liquid chromatography (UHPLC) system coupled with the ^1^D quaternary pump HPLC (or UHPLC) system. The system could not utilize newer quaternary UHPLC systems for the second dimension, since among other things, the software only allowed two solvent channels to be configured for the ^2^D in 2D-LC.

More separation options were needed than were commercially available because milk triacylglycerols (TAGs) are very complex and could not be adequately separated using 1D-LC, due to the presence of numerous isobaric isomers. Also, milk TAGs contain a large number of very short-chain fatty acids (SCFAs), down to C4, that were not retained well on the conventional C18 columns normally used for TAG analysis. Furthermore, the SCFAs are saturated, so the TAGs that contained them were not separated using the silver-ion UHPLC used as the ^2^D to separate unsaturated TAGs, especially those containing *trans* double bonds [1, 2].

To overcome these limitations, we employed two contact closure (CC) controlled 4-port, 2-position valves (4P2PVs) that were joined together to emulate the eight-port switching valve on the commercial 2D-LC system. To automate the CCs, the 4P2PVs were connected to a timed contact closure circuit (TCCC) that provided consistent timed CCs to switch the 4P2PVs uniformly throughout the chromatographic separation. Control of the TCCC was incorporated into the wireless communication contact closure system (WCCCS) that we previously reported [3]. Using this prototype instrument configuration we were able to perform separations employing three LC systems and four mass spectrometers (LC3MS4), in which the ^1^D HPLC separation was monitored using electrospray ionization mass spectrometry (ESI-MS) and atmospheric pressure chemical ionization (APCI) MS in parallel (= LC1MS2), and two ^2^D UHPLC separations were conducted simultaneously in parallel, with a binary UHPLC using a silver-ion column monitored using atmospheric pressure photoionization (APPI) MS (= LC1MS1) and a quaternary UHPLC using a C8 column monitored using ESI-MS (= LC1MS1). Thus, we report here the hardware and software components necessary to accomplish 2D-LC having two parallel second dimensions, with detection by four mass spectrometers simultaneously in parallel, plus 7 other detectors, for LC1MS2 × (LC1MS1 + LC1MS1) = LC3MS4. This new experimental arrangement provides a new tool to allow us to conduct method development for complex lipid analysis.

## Main Text

### Materials and methods

**Fig. 1:**
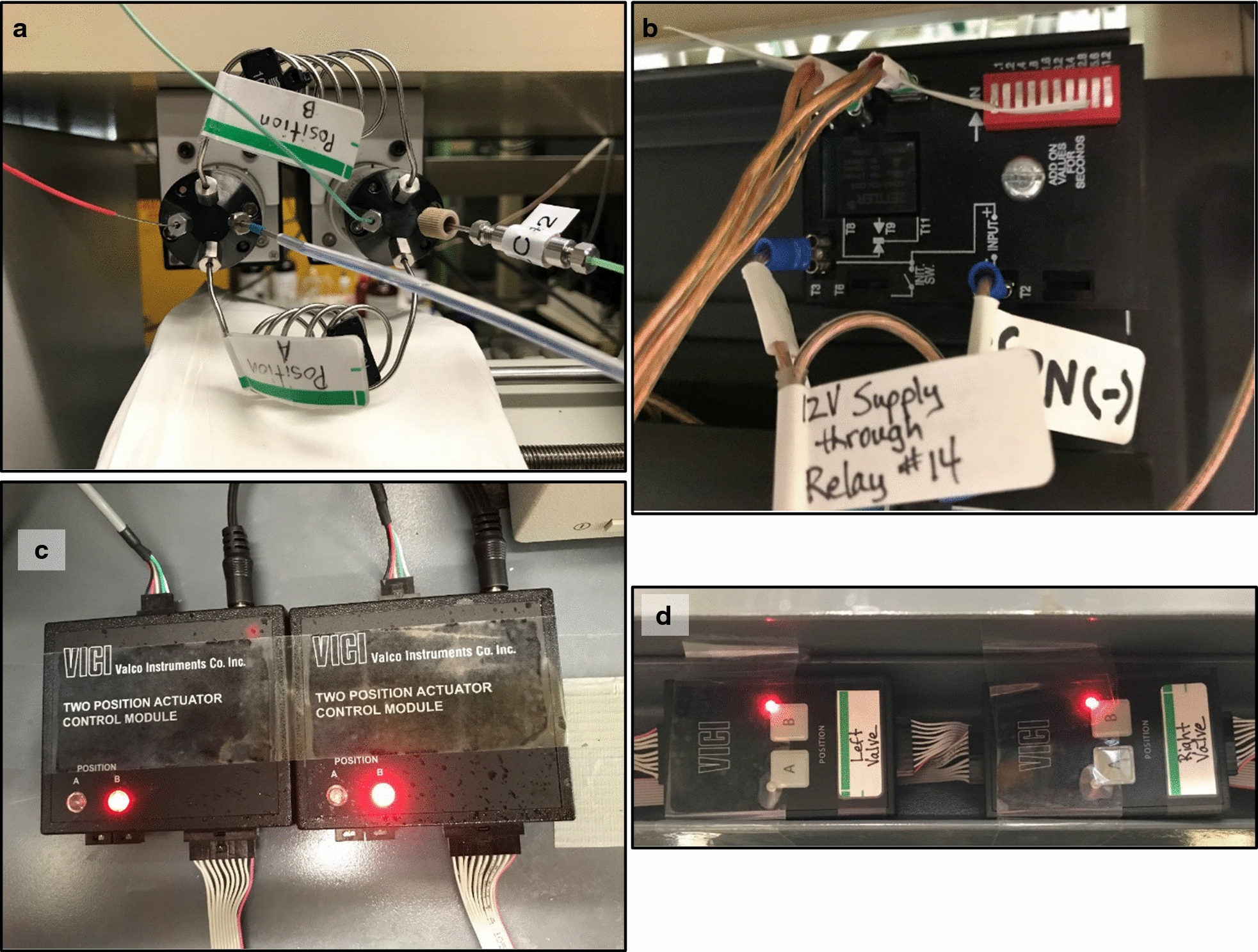
Components of the timed contact closure circuit (TCCC) controlled dual switching valve system. **a** two four-port two-position high-pressure valves joined by sample loops; **b** TCCC receives activating voltage from relay #14 in wireless communication contact closure system, with DIP switch set to 57.3 s = 1011110001; **c** two-position actuator control modules; **d** manual valve controllers

**Fig. 2:**
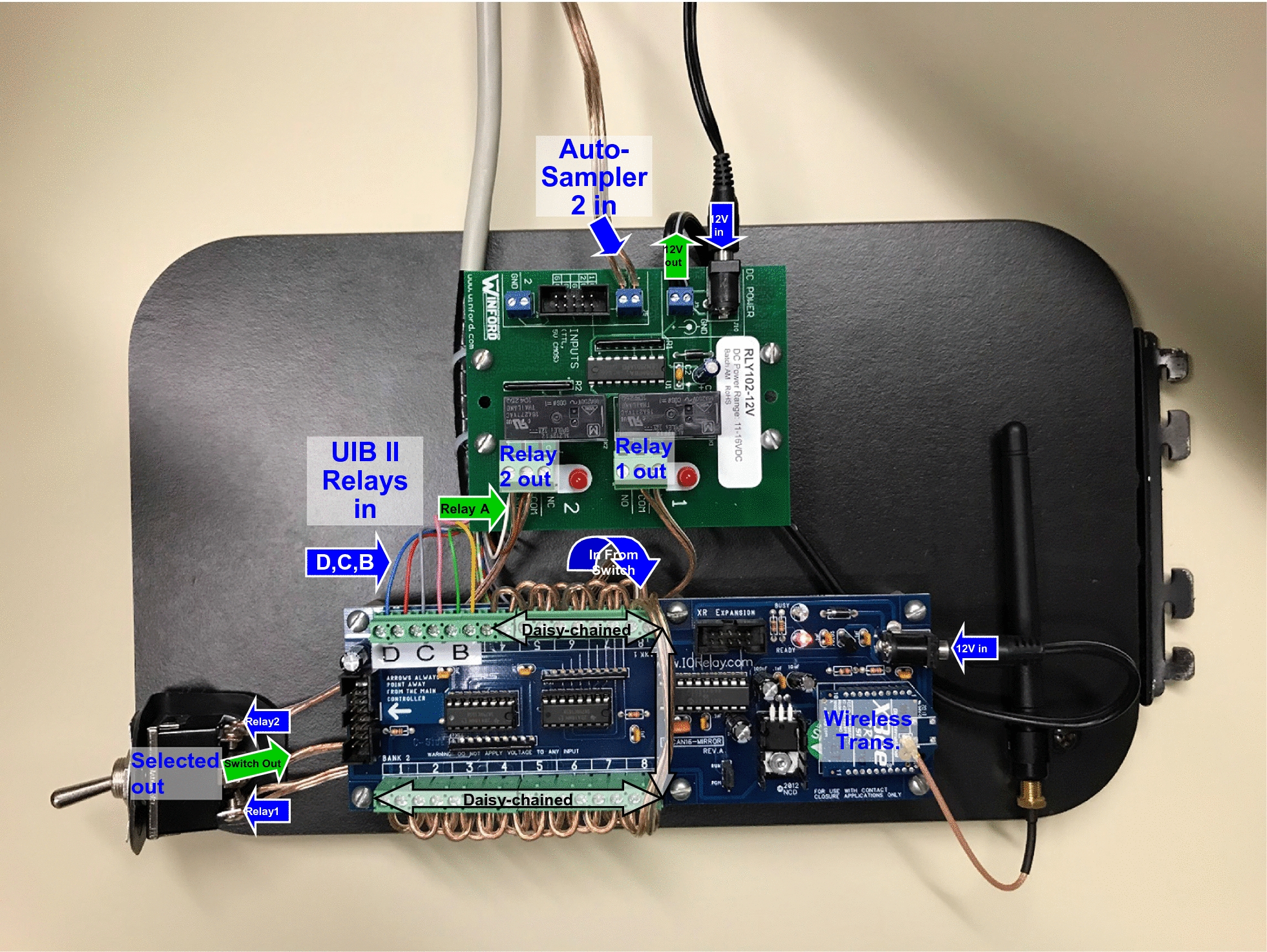
Updated relay board and wireless communication contact closure system (WCCCS) sender board #2, attached to the Agilent 1290 Infinity Flex II autosampler and Universal Interface Box II (UIB II) relays (A-D). The top (smaller) board was a relay board to convert the 5 V TTL signal from the autosampler to a simple contact closure (CC). Relays from the UIB II were simple CCs, so were directly connected to the relay board output (relay A) and WCCCS sender inputs (relays B-D). The switch on the left allowed selecting autosampler or Relay A as the start signal; the selected output is daisy-chained from channels 1 to 13. Relay B was sent to the WCCCS switchable channel 14, Relay C to channel 15, and Relay D to channel 16. All WCCCS signals were switchable between the Agilent 1200 HPLC and the Agilent 1290 Infinity Flex II UHPLC via a switch manifold

**Fig. 3:**
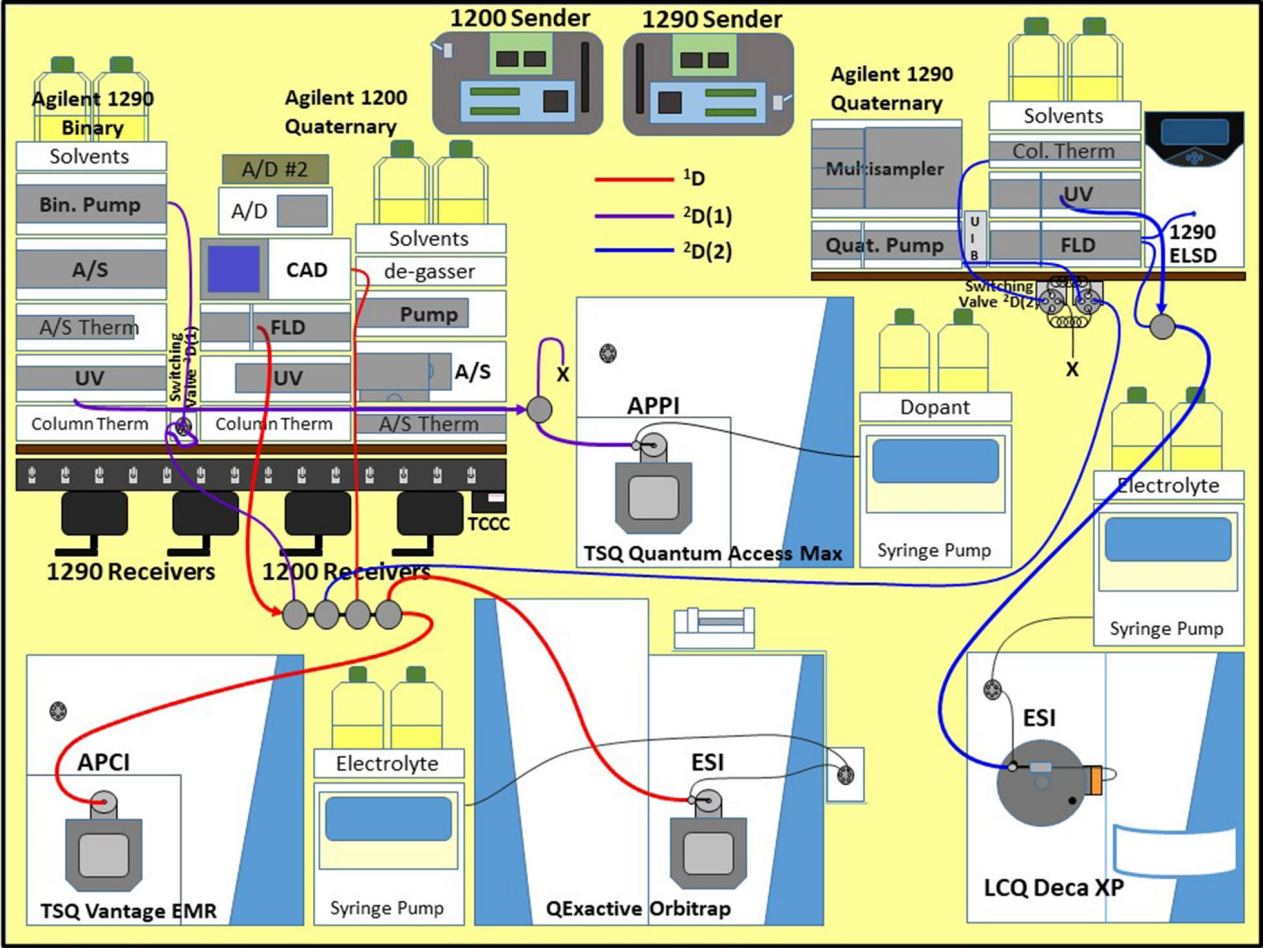
Arrangement of instruments, wireless communication contact closure system (WCCCS) (with sender boards, receivers, and switchable contact closure distribution manifold) and timed contact closure circuit (TCCC) for comprehensive two-dimensional liquid chromatography (2D-LC) with two parallel second-dimensions [^2^D(1) and ^2^D(2)], for LC1MS2 × (LC1MS1 + LC1MS1) = LC3MS4. The Agilent 1200 injected sample into the ^1^D non-aqueous reversed-phase HPLC system with all flow directed through UV and fluorescence (FLD) non-destructive detectors, then to the splitter composed of Valco tees and fused-silica capillaries to other detectors and switching valves (SVs). In addition to UV and FLD the ^1^D was monitored by a corona charged aerosol detector (CAD) (collected by two A-to-D converters), a tandem sector quadrupole (TSQ) mass spectrometer operated in atmospheric pressure chemical ionization (APCI) mode, and a high-resolution accurate-mass QExactive orbitrap instrument in electrospray ionization (ESI) mass spectrometry (MS) mode with NH_4_OCOH electrolyte, for five detectors in the ^1^D. The SV(1) attached to the Agilent 1290 binary UHPLC system loaded fractions onto a silver-ion column, followed by UV detection, then detection by atmospheric pressure photoionization (APPI)-MS on a TSQ instrument (with acetone dopant added via syringe pump). SV(1) was controlled by the Agilent OpenLab ChemStation (OLCS) 2D-LC software. SV(2) loaded sample onto the Agilent 1290 quaternary UHPLC system with C8 column, followed by detection by UV, then splitting to go through another FLD and to an evaporative light-scattering detector (ELSD) and the other branch going to an LCQ Deca XP ion trap mass spectrometer in ESI-MS mode with NH_4_OCOH electrolyte. The ^2^D SV(2) was controlled by the TCCC via the WCCCS, with a manually programmed shifted gradient in the OLCS software

Two Cheminert nanovolume 4P2PVs with microelectronic actuator (#C85U-6674EMT, Valco Instruments Co., Inc., Houston, TX, USA) rated to 20,000 psi (1379 bar) were joined together as shown in Fig. 1a. The microactuators for the two valves were both connected to a 12 V 10A 10-bit DIP-switch-controlled Infitech binary digital power timed delay relay (#BRKR1A411, Infitech, Inc., Syracuse, NY, USA) having a time delay range of 0.1 to 102.3 s as shown in Fig. 1b. The 12 V power required to initiate the on/off recycling of the TCCC was wired through relay #14 of the previously reported WCCCS receiver boards (Ref. 3 Figures 2B, 3). Both WCCCS sender boards were rewired to add individual controls of relays B, C, and D, as shown in Fig. 2. Relay A was already wired through the single switch on the sender board mounting units, to provide a timed relay as a longer start signal. Switch #14 on the switch distribution manifold made the voltage to the TCCC from the WCCCS switchable between Relay B from the Agilent 1200’s G1329A autosampler or from the Agilent 1290 Infinity Flex II’s Universal Interface Box II (UIB II), either of which was controlled by the relay control timetable in respective versions of OpenLab Chemstation (OLCS) C.01.09 software running on both systems.

Repetitive CCs from the TCCC in Fig. 1b were connected to the microelectric actuator control modules (MACMs) shown in Fig. 1c. The CC connections to the two MACMs were joined together to ensure they activated simultaneously, and connected to the horizontal and one vertical spade terminal on the TCCC, as shown as in Fig. 1b. Because both connections were connected to one of the two vertical terminals, the time set on the DIP switch was one-half of the valve cycle time. The maximum setting of the model of TCCC demonstrated here was 102.3 s, so the TCCC could be used for cycle times up to 204.6 s (3.41 min). ^2^D separations had run times of 1.91 min (=114.6 s), so the DIP switch was set to 57.3 s, or binary 1000111101.

The same mass spectrometers and other detectors that were used for the previous reports on LC2MS4 were used for the ^1^D and ^2^D(1) [2, 4]. Detailed descriptions of the instruments and parameters used for comprehensive 2D-LC with quadruple parallel MS (LC2MS4 = LC1MS2 × LC1MS2) were given previously (see Supplemental Materials to Ref. 4). The Agilent Infinity Flex II quaternary UHPLC has been added since that report, and is used for the ^2^D(2) separation. The exact same parameters that were used previously were used for the UV and fluorescence (FLD) detectors on the new UHPLC system [4]. The evaporative light scattering detector (ELSD) (G4261B, Agilent Technologies, Santa Clara, CA, USA) was moved from the monitoring the ^1^D to monitoring the ^2^D(2). The overall arrangement of all liquid chromatographs, auxiliary detectors, and mass spectrometers is depicted in Fig. 3. The arrangement of branches from the Valco tee flow splitting system previously used [4] was modified slightly to provide flow to the ^2^D(2). A 75 μm i.d. × 2.5 m long piece of fused silica capillary (#160-2644-10, Agilent Technologies, Inc., Santa Clara, CA, USA) was connected to a Valco union via an adapting sleeve (#F-242X, IDEX Health and Science, LLC, Oak Harbor, WA, USA), with a 0.10 mm i.d. x 10 cm piece of stainless steel tubing on the distal end to attach to the switching valve (seen at the far right in Fig. 1a). The capillary length produced a flow rate of 53.6 μL/min, so that the fill time of the alternating 100 mL sample loops was 1.86 min, with a total run time (= modulation time) of 1.91 min, to exactly match the commercial 2D-LC system. Another Valco tee splitter was added after the ^2^D(2) UV detector (Fig. 3), to split flow between the FLD + ELSD and the LCQ Deca XP ion trap mass spectrometer operated in ESI-MS mode.

### Results and discussion

There were several limitations imposed by the OLCS software that had to be worked around to enable the ^2^D(2) separation that we desired. First, the 2D-LC arrangement of instruments that was commercially available only allowed a binary UHPLC pump to act as the ^2^D separation. Therefore, the quaternary UHPLC (LC#3) was controlled separately and attached to the 4P2PVs to allow the ^2^D(2) separation, while the Agilent 1290 binary UHPLC (LC#2) was used for the ^2^D(1). Second, to accomplish the same type of shifted gradient that was allowed on the commercial 2D-LC system, each individual sub-gradient had to be manually programmed into the quaternary pump timetable. Unfortunately, the OLCS software has a limit of 100 time steps allowed in gradient timetables. Each sub-gradient was composed of five time points: (1) start initial isocratic composition, (2) end of isocratic composition and start of gradient, (3) end of gradient and start of hold composition, (4) end of hold composition and start of recycle gradient, and (5) end recycle to next isocratic composition and held until start of next sub-gradient. Therefore, the 100 allowed steps divided by 5 steps per sub-gradient permitted 20 sub-gradients per UHPLC method. Each sub-gradient was set to 1.91 min to exactly match the shifted gradient times from the commercial 2D-LC binary UHPLC. This was not required, but allowed simplified data analysis using identical parameters to those used for the ^2^D(1) in the LC x LC software (GC Image, Inc., Lincoln, NE, USA). Nevertheless, the OLCS software allowed only 20 sub-gradients that were each 1.91 min, for a total method time of 38.2 min. Therefore, two different methods with ≤ 100 steps each were required to cover the full time used for the ^1^D separation.

The previously reported [2, 4] first-dimension non-aqueous reversed-phase (NARP)-HPLC (LC#1) separations of TAGs were shortened to 76.4 min, to exactly match two 38.2 min ^2^D separation methods on the ^2^D(2) UHPLC. The 54 min separation of fat-soluble vitamins (FSVs) was eliminated, since natural cow’s milk is not fortified and does not contain the early-eluting vitamin D that we have analyzed in other samples. To span the length of the ^1^D NARP-HPLC separation, two different methods were programmed into the quaternary UHPLC (LC#3) OLCS control software, and these were joined together into a “sequence”. The first method in the sequence used the injection parameter “manual injection”, for which the start was triggered using Relay C of the Agilent 1200 HPLC (^1^D, LC#1) attached to the WCCCS sender board #1 (wired identically to that shown in Fig. 2). The second method in the sequence used the injection parameter “no injection”, which started the second method immediately after the end of the first method. Relay A, attached to WCCCS sender board #1 (wired to the autosampler in LC#1 as shown in Fig. 1a in an earlier report [3] and identical to Fig. 2) started the other detectors and components for the ^1^D (LC#1) and ^2^D(1) (LC#2), while relay A from LC#3 attached to WCCCS sender board #2 started the detectors for the ^2^D(2). We chose to use a separate relay, Relay C, to start the Agilent 1290 Infinity Flex II (LC#3) from the Agilent 1200 (LC#1) to allow more flexibility, e.g., to start the ^2^D(2) at a different time to analyze only a sub-section of the ^1^D separation, if desired. Since this report describes the hardware and software components necessary to automate the ^2^D(2) separation, full details of the complete set of LC gradient parameters will be described in reports of the application of this new system. At the beginning of an experiment, the ^2^D(2) instrument (LC#3) was started (“Run Sequence”) and all four mass spectrometers were started (via various versions of Xcalibur software), and all waited for the contact closure start signal from the 2D-LC Agilent 1200 autosampler (LC#1), via the WCCCS.

Using the reported combination of instruments, WCCCS (with sender boards, receivers, and switchable contact closure distribution manifold), TCCC, and CC-controlled ultra-high pressure valves, we were able to accomplish the first example of the hardware and software necessary for comprehensive 2D-LC with two parallel second-dimensions [^2^D(1) and ^2^D(2)], for LC1MS2 × (LC1MS1 + LC1MS1) = LC3MS4. This new configuration of instruments and CC control hardware and software allows new types of automated multi-dimensional liquid chromatography for greater separation of complex samples.

## Limitations

This work requires a moderate level of mechanical aptitude to accomplish construction of these components. This work requires the availability of three liquid chromatographs and four mass spectrometers.

## Data Availability

Not applicable.

## Abbreviations

^1^D: first dimension
^2^D: second dimension
2D-LC: two-dimensional liquid chromatography
4P2PV(s): 4-port, 2-position valve(s)
APCI-MS: atmospheric pressure chemical ionization mass spectrometry
APPI-MS: atmospheric pressure photoionization mass spectrometry
CC(s): contact closure(s)
ELSD: evaporative light scattering detector
ESI-MS: electrospray ionization mass spectrometry
FLD: fluorescence detector
FSV(s): fat-soluble vitamin(s)
HPLC: high performance liquid chromatography
MACM(s): microelectric actuator control module(s)
NARP: non-aqueous reversed-phase
OLCS: OpenLab Chemstation
SCFA(s): short-chain fatty acid(s)
TAG(s): triacylglycerol(s)
TCCC: timed contact closure controlled circuit
UHPLC: ultra-high performance liquid chromatography
UIB: universal interface box
WCCCS: wireless communication contact closure controlled system
